# Supplementary material for: The Genetic Architecture of Milling Quality in Spring Oat Lines of the Collaborative Oat Research Enterprise
Source: Foods. 2021 Oct 16;10(10):2479. doi: 10.3390/foods10102479 (PMC8535619; doi:10.3390/foods10102479)

Supplemental Figure S1. The phenotypic distributions of 6 milling quality-related traits averaged across location years.

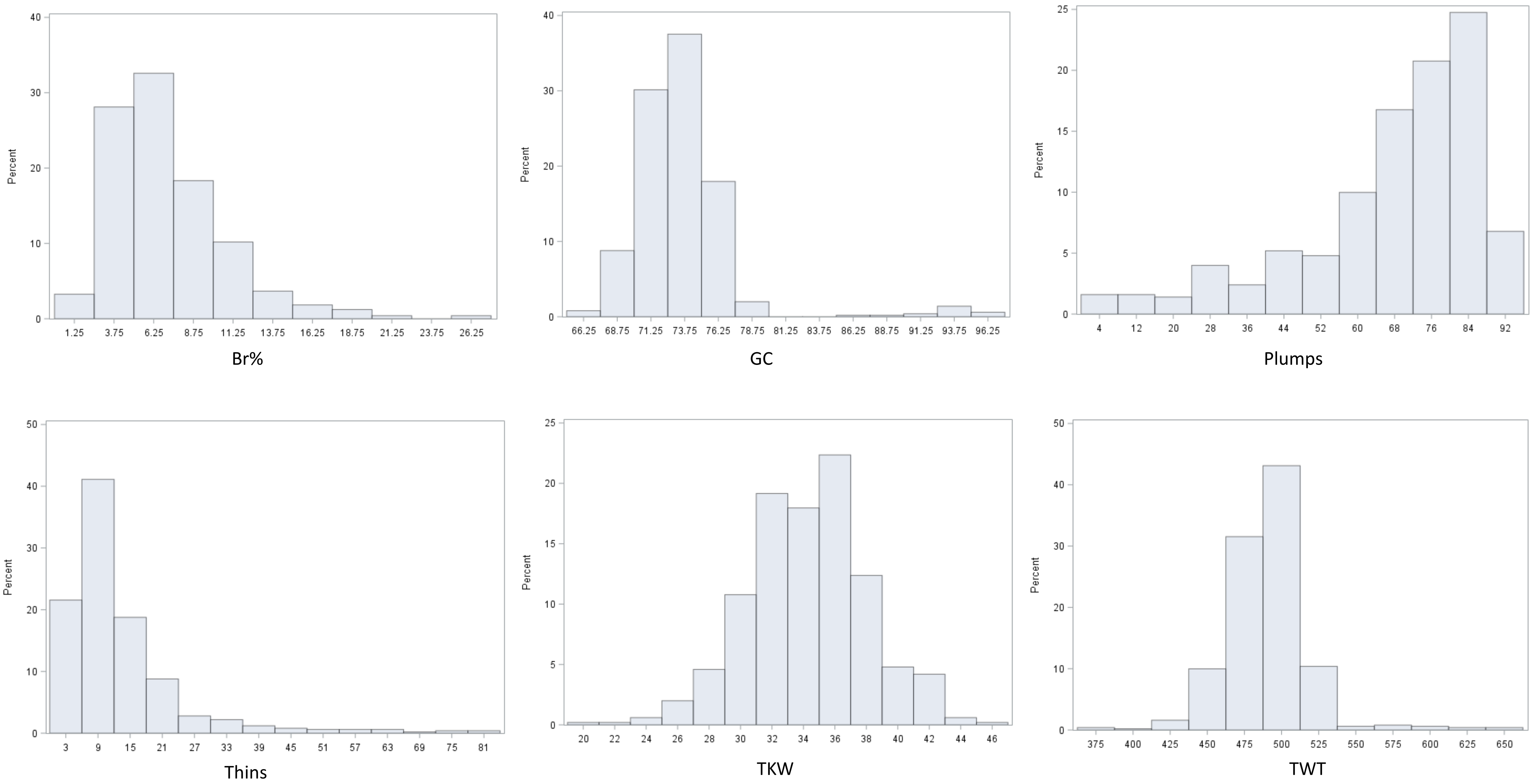

Supplement: Supplementary file 1 [file foods-10-02479-s001.zip › foods-1396795-supplementary.pdf]
